# Synthetic Control Methods for the Evaluation of Single-Unit Interventions in Epidemiology: A Tutorial

**DOI:** 10.1093/aje/kwab211

**Published:** 2021-08-03

**Authors:** Carl Bonander, David Humphreys, Michelle Degli Esposti

**Keywords:** causal inference, internal validity, panel data, program evaluation, quasi-experiments

## Abstract

Evaluating the impacts of population-level interventions (e.g., changes to state legislation) can be challenging as conducting randomized experiments is often impractical and inappropriate, especially in settings where the intervention is implemented in a single, aggregate unit (e.g., a country or state). A common nonrandomized alternative is to compare outcomes in the treated unit(s) with unexposed controls both before and after the intervention. However, the validity of these designs depends on the use of controls that closely resemble the treated unit on before-intervention characteristics and trends on the outcome, and suitable controls may be difficult to find because the number of potential control regions is typically limited. The synthetic control method provides a potential solution to these problems by using a data-driven algorithm to identify an optimal weighted control unit—a “synthetic control”—based on data from before the intervention from available control units. While popular in the social sciences, the method has not garnered as much attention in health research, perhaps due to a lack of accessible texts aimed at health researchers. We address this gap by providing a comprehensive, nontechnical tutorial on the synthetic control method, using a worked example evaluating Florida’s “stand your ground” law to illustrate methodological and practical considerations.

## Abbreviations:

COVID-19: coronavirus disease 2019
SCM: synthetic control method

Social interventions, such as national policies, laws, or changes to the physical environment, hold the promise of impact on the health of populations with minimal individual effort (1, 2). However, it is challenging to evaluate social interventions using conventional methods for causal inference, especially in data with few units (e.g., states, countries). Randomization requires large samples to achieve equivalence between groups, and regression-based methods (e.g., propensity score estimation) can perform poorly in small samples (3).

The main challenge is estimating what would have happened without the intervention in a specific region or population group. Would homicide rates in Florida be different without their “stand your ground” law (4)? Would Jena, Germany, have had a more severe coronavirus disease 2019 (COVID-19) outbreak without early face-mask regulations (5)? Answering these questions credibly usually requires similar but unexposed controls.

Controlled before-after studies are often used to evaluate social interventions (6). With repeated outcome measurements from the treated unit and controls, these designs can control for all confounders that do not vary over time. However, to produce valid results, the control(s) and the treated unit must share the same outcome trend (7). Similarity on covariates that are strongly predictive of future changes in the outcome can also be important (8).

In settings with few units, none of the available controls may be sufficiently similar to provide a suitable comparison for the treated unit. The synthetic control method increases the possibility of finding a good match by considering weighted combinations of units, also known as “synthetic controls” (9). Unlike inverse propensity for treatment weighting (10), synthetic control weights are calculated through optimization instead of propensity scores, which avoids small sample bias from estimating the propensity score based on only one treated unit (3).

**Figure 1:**
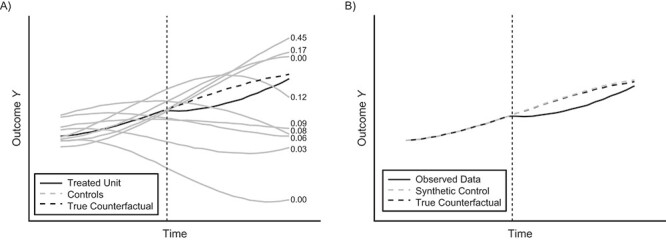
Simulated example with a known intervention effect. A) Raw data from a balanced panel data set with time series of an outcome *Y* from a single treated unit and several potential controls. Given this data, the synthetic control method determines the unit weights that generate the best-fitting synthetic control unit in the pre-intervention period (before the dashed vertical line). Each control unit is assigned a unit weight ranging from zero to 1 (the sum of the weights is always 1); the numbers on the right-hand side of the plot area reflect these weights. B) The synthetic control outcomes are then given by the weighted sum of the outcomes among controls, which are obtained by multiplying the time-specific outcomes in each unit with its respective unit weight, and then summing across all control units. The time series of postintervention outcomes in the synthetic control provide an estimate of the counterfactual outcomes in the treated unit, which is then compared with the observed data to estimate the intervention effect.

The method was introduced in 2003 by Abadie and Gardeazabal (11) and later formalized by Abadie et al. (9, 12). Since then, it has received considerable attention in the statistical literature (3, 13), and Athey and Imbens called it “the most important innovation in the policy evaluation literature in the last 15 years” (6, p. 9). Despite their potential to enhance the evaluation of social interventions and other health policies (5, 14–18), synthetic controls are reportedly underused in health research compared with the social sciences (19). We believe that one reason may be that most texts detailing the method are aimed at a technical audience or social scientists, which may hamper understanding of its potential uses and pitfalls in epidemiologic research. We address this gap by offering an accessible introduction and comprehensive guide to synthetic control methods aimed at applied epidemiologists.

The tutorial is organized as follows. The following section provides an overview of the synthetic control methodology. The subsequent sections provide details on its strengths and limitations, data requirements, quality assessment, and effect estimation procedures, respectively. The final section discusses ways of handling practical and methodological problems. We use data from an evaluation of Florida’s “stand your ground” law, enacted in October 1, 2005, to illustrate estimation practices and methodological considerations (4, 15). (The law extends the right to use lethal force in self-defense to public places when threat is perceived.) The data and code to reproduce our analyses is available online (20).

## OVERVIEW OF THE SYNTHETIC CONTROL METHOD

The synthetic control method (SCM) is an analytical approach that can be applied in controlled before-after studies. Controlled before-after designs use panel data—repeated measurements of an outcome variable (e.g., homicide rates) in multiple units (e.g., US states) over time—to evaluate the effects of an intervention, event, or policy by comparing outcomes in exposed versus unexposed units (treated and control units, respectively). These designs require comparable control units. For example, if an intervention is delivered only in Florida due to a state-specific policy change, then the remaining US states without similar policies may serve as potential control units. Alone these unexposed states may not provide good matches for Florida on characteristics that may confound the association between the intervention and the outcome (e.g., systematic differences in trends in homicide rates, and other factors such as climate, population density, levels of poverty, and crime), but combined they can better approximate Florida and potentially control for such confounding. SCM exploits this observation by weighting and combining information from a set of potential control units into a “synthetic control unit” that better matches the treated unit in the pre-intervention period (9).

SCM uses an optimization algorithm tailored specifically for panel data with a single treated unit and multiple potential control units. We provide further details below but illustrate the general idea in Figure 1. In Figure 1A, we have used the algorithm to construct a synthetic control unit using time-series data from a single treated unit and a set of control units. Its objective is to construct a synthetic control that best matches the treated unit in terms of trends in the outcome (and other covariates) during the pre-intervention period to control for differences in pre-intervention characteristics and time trends. The resulting synthetic control unit, illustrated in Figure 1B, is then used to estimate the counterfactual outcomes (i.e., what would have happened in the absence of the intervention).

## WHEN IS THE SYNTHETIC CONTROL METHOD APPROPRIATE?

SCM was developed for evaluating interventions that occur at the aggregate level, in a distinct unit (e.g., a state, country, age group), and a clearly differentiated point of time (12). For example, a state-specific change to the regulation of opioids is a policy intervention that occurs in a discrete geographical region (i.e., the state) with an implementation time point (i.e., the passing of the policy or legislation). This specificity allows evaluators to define exposed and unexposed geographical regions as treated and potential control units, respectively, and define the pre- and postintervention period. Not all interventions have exposed and unexposed regions or a clear starting point. For example, the COVID-19 pandemic raises difficult questions about which regions are affected by the “intervention” and when they were affected due to diffuse and gradual contamination. In this case, SCM may not be the most appropriate methodology.

### Advantages of the synthetic control method over alternative evaluation methods

SCM has 3 main strengths. First, it provides data-driven and formal criteria for selecting controls, which may reduce researcher bias compared with manually selecting control unit(s) (21). Second, it may help reduce the risk of bias in policy evaluations. Its potential benefits can be easily seen within the context of the difference-in-differences framework (7), which is a widely used analytical approach in controlled before-after studies of social interventions (6). Like synthetic controls, difference-in-differences uses panel data on exposed and unexposed groups or units (e.g., regions), but valid difference-in-differences estimation relies on the assumption that both groups’ outcomes would have followed the same trend in absence of the intervention (the parallel trends assumption). While the assumption cannot be directly assessed, a standard validity check is to test for between-group differences in pre-intervention trends in the outcome (21). SCM formally incorporates this idea by reweighting the controls to match on pre-intervention trends in the treated unit, thus increasing the likelihood that the parallel trends assumption holds (22). Third, SCM offers estimates of the shape of the effect over time as it constructs a time series for the synthetic control unit for the full postintervention period (9). This is an advantage over another popular alternative for evaluation of social interventions, the interrupted time-series design, which requires making prespecified modeling assumptions about the shape of the intervention effect over time (i.e., an impact model) (23).

Since its initial development, SCM has also become increasingly flexible. Different estimation strategies and generalizations have been proposed to accommodate a variety of data settings, including more flexible estimation strategies for settings with one treated unit (17, 24–26), multiple treated units (27–31), and staggered adoption dates (22, 32, 33) (Table 1). This paper focuses on the original version of the method, but interested readers may consult Table 1 and associated references for more information about alternative approaches.

**Table 1:** Estimation Strategies for Synthetic Controls and Similar Designs Under Different Data Settings

**Estimation Strategy** ^**a**^	**Data Setting** ^**a**^		
	**One Treated Unit, Several Potential Controls**	**Many Treated Units With the Same Adoption Date, Many Controls**	**Several Treated Units With Staggered Adoption Dates**
Balancing^b^	The (canonical) synthetic control method; elastic net regression; Bayesian structural time-series modeling	Propensity score weighting based on covariates and pre-intervention outcomes; synthetic control methods for micro-level data; trajectory balancing	Synthetic control method applied separately to each treated unit (pooled estimates)
Outcome modeling^c^	Interactive fixed effects regression (“generalized synthetic control method”)	Difference-in-differences (matching on pre-intervention trends); interactive fixed-effects regression (“generalized synthetic control method”)	Interactive fixed effects regression (“generalized synthetic control method”)
Doubly robust estimation^d^	The augmented synthetic control method	Synthetic difference-in-differences; penalized synthetic control method for disaggregated data	Pooled augmented synthetic control method with staggered adoption

^a^ The estimators are categorized according to typical data setting and estimation strategy. Each cell contains the name of a method. (See text for references; the table is not intended to be exhaustive.)

^b^ Balancing refers to estimators that use weights to achieve balance on pre-intervention outcomes and (if applicable) covariates.

^c^ Outcome modeling refers to strategies that directly model postintervention outcomes (e.g., regression).

^d^ Doubly robust estimation refers to methods that combine both strategies.

### Contextual sources of bias

Even if a synthetic control closely matches the treated unit, contextual sources of bias also need to be considered when determining the appropriateness of SCM. These are the same biases that can affect most controlled before-after studies, including: 1) impacts on pre-intervention outcomes due to the anticipation of the intervention before the intervention is officially implemented (34); 2) impacts on control regions (35) (i.e., contamination/spillover effects); and 3) co-interventions or other postintervention events that do not have equivalent impact on the outcomes in the synthetic control and treated unit, confounding the effect of the intervention of interest (15). We further discuss bias related to statistical issues below.

## DATA REQUIREMENTS

### The outcome(s)

There are no strict requirements on the outcome variable for using SCM other than that it should be (approximately) continuous, and repeated measures for the outcome must be available (see the next section). The method can handle outcomes with fixed upper or lower bounds (e.g., nonnegative count data) (17). This means that most aggregate epidemiologic measures, such as mortality counts, rates, or prevalences, should be compatible with SCM. However, some aspects of the outcome may influence how SCM is most appropriately employed. We discuss these issues further below.

The outcome data in our example is monthly homicide rates (for all ages) spanning from January 1999 to December 2014, which we obtained at the state level from the Centers for Disease Control and Prevention’s Wide-Ranging Online Data for Epidemiologic Research database.

### Temporal information

SCM requires sequential measures in the outcome before and after the intervention in both the treated unit and pool of potential control units in the form of a balanced panel data set, which means that all units in the data need to be observed over the same time period (e.g., 1999–2014) without any missing values within that period. There are no fixed limits for the number of data points required in the pre- or postintervention period, which is a product of the time period and time intervals of measurement (e.g., days, months, years). The method can be applied with only one pre-intervention time point, but it is usually more credible if it can be shown that the synthetic control matches the treated unit on outcome trends in a longer pre-intervention period (9).

We note 2 contextual factors to consider when deciding on an appropriate study period: 1) Events or interventions in the “pre” period that vastly change the characteristics of the outcome (e.g., its trend or level) may warrant the use of a shorter time window than the full available data period, as such events may not be desirable to match on; and 2) if the expected effect is gradual or delayed, the “post” period needs to be sufficiently long for the effect to have time to manifest (3).

### Covariates

The use of covariates is optional (8), but including a set of covariates that are predictive of the postintervention outcomes in absence of the intervention can potentially improve causal inference (3, 8), especially if the pre-intervention period is too short to match on underlying trends using outcome data alone. The covariates may be time-invariant or time-varying (in the latter case, data from each time point can be entered as a separate covariate to enable SCM to match on covariate trends).

The covariates are typically pre-intervention characteristics that are hypothesized to affect the postintervention outcomes that would have been realized in absence of the intervention (e.g., risk factors or sociodemographic factors). The appropriateness of covariates can, for instance, be assessed using graphical methods, such as logic models and directed acyclic graphs (36), in combination with subject-matter knowledge. As an illustration, we present a logic model for our analysis of Florida’s “stand your ground” law in Web Figure 1 (available at https://doi.org/10.1093/aje/kwab211), which we used to determine appropriate covariates. Detailed information about the data is presented in Web Table 1. Briefly, we consider unemployment rates, Republican voters, urbanicity, alcohol consumption, firearm ownership rates, age and racial composition, violent crime rates, and incarceration rates as covariates. The SCM algorithm contains an automated determination of variable importance that prioritizes a good match on strong predictors of the untreated potential outcomes over weak predictors as they are assumed to give rise to a more convincing synthetic control (9). This algorithm further allows for data-driven selection of appropriate covariates.

### The donor pool

SCM requires a set of unexposed units to make up a suitable “donor pool” of potential controls. Control units in the donor pool are described as potential controls because being included in the donor pool does not mean that the algorithm will include them in the synthetic control unit.

Although there must be more than one potential control unit to construct a weighted average, there are no other fixed specifications for the number of potential control units needed in the donor pool. A suitable donor pool should comprise units that: 1) share the same definition of a unit as the treated unit (e.g., distinct geographical region); 2) are not exposed to the intervention (or any similar intervention) during the study period; and 3) do not experience other isolated events that cause large temporary shocks during the pre-intervention period that are not predictive of the postintervention outcomes (e.g., a terrorist attack) (3, 9).

In our example, we included all 15 US states (i.e., equivalent geographical regions to Florida) that did not enact similar laws (i.e., are unexposed) during the study period: Arkansas, Connecticut, Delaware, Hawaii, Iowa, Maine, Maryland, Massachusetts, Nebraska, New Jersey, New York, North Dakota, Ohio, Rhode Island, and Wyoming. To avoid matching on a large temporary shock, we also excluded deaths caused by the 9/11 terrorist attack from the September 2001 data in New York.

## CONSTRUCTING THE SYNTHETIC CONTROL UNIT

SCM uses optimization to determine the best set of weights for the controls given the available data. Software for running the optimization is available for Stata (StataCorp LLC, College Station, Texas), R (R Core Team, R Foundation for Statistical Computing, Vienna, Austria), and MATLAB (MathWorks, Inc., Natick, Massachusetts) (9). In our example, we use the original “Synth” package for R in combination with the “Multivariate Synthetic Control Method Using Time Series” package, the latter of which runs a more numerically stable optimization (37, 38). For technical details, see Abadie et al. (9) and Abadie and Gardeazabal (11).

The optimal weights ${w}_i^{\ast }$ are determined by minimizing the distance between the synthetic control and the treated unit using a variable importance–weighted mean squared error function (9, 12). The weights are constant across the study period and constrained to be nonnegative and sum to 1. The constraints restrict the method to only allow for estimates based on interpolation within the empirical distribution of the data among controls (9, 24) (details below). The function that SCM aims to minimize can be expressed as:

(1) \begin{equation*} \sum \limits_{k=1}^K{v}_k{\left({X}_{1k}-\sum \limits_{i=2}^N{X}_{ik}{w}_i^{\ast}\right)}^2, \end{equation*}

where each variable *k* is assigned an importance weight ${v}_k$; ${X}_{1k}$ represents the value of the variable *k* in the treated unit (indexed by *i* = 1), and ${X}_{ik}$ represents the values among controls, which, when summed together with the weights ${w}_i^{\ast }$, give rise to the distribution of the variables in the synthetic control unit. The variables in the matching vector $\mathbf{X}$ are the ones that SCM will try to match on, which means that the weights are determined so that the weighted average, ${\sum}_{i=2}^N{X}_{ik}{w}_i^{\ast }$ in equation 1, is as similar as possible to ${X}_{1k}$ given the variable weights ${v}_k$. The contents of $\mathbf{X}$ can be the value of the outcome variable at each pre-intervention time point, some combination of pre-intervention outcomes (e.g., the average of the pre-intervention outcomes, a vector of moving averages, etc.), and other covariates (9) (see Covariates, above).

The function of the variable weights ${v}_k$ is to assign higher priority to strong predictors of the outcome variable, allowing for greater imbalance on weak predictors (9). This feature allows for more effective use of the available data, as a perfect balance can typically not be achieved on all variables in small samples (11). The variable weights can be manually specified (e.g., all variables can be given equal weight or assigned a relative importance weight based on previous research). However, the typical approach in SCM is to use a data-driven subroutine to estimate the strength of the correlation between the variables entered into the matching procedure and the pre-intervention outcomes in the treated unit (11).

**Table 2:** The Unit Weights That the Synthetic Control Method’s Optimization Algorithm Assigned to Each Control State to Construct a Synthetic Florida for Evaluating Florida’s “Stand Your Ground” Law

**State**	**Unit Weight**
Arkansas	0
Connecticut	0
Delaware	0.02
Hawaii	0.03
Iowa	0
Maine	0
Maryland	0.26
Massachusetts	0.12
Nebraska	0
New Jersey	0.14
New York	0.35
North Dakota	0
Ohio	0
Rhode Island	0.08
Wyoming	0

**Table 3:** Covariate Balance Between Florida, Synthetic Florida, and the Unweighted Sample Average of All 15 States in the Donor Pool, Before the Implementation of Florida’s “Stand Your Ground” Law, January 1999–September 2005

	**Florida** ^**a**^						**All Control States** ^**b**^			
	**Real**			**Synthetic**						
**Variable**	**Mean**	**%**	**Rate** ^**c**^	**Mean**	**%**	**Rate** ^**c**^	**Mean**	**%**	**Rate** ^**c**^	**V**
State-level covariates^d^										
Ethanol consumption^e^	2.92			2.45			2.60			0
Unemployment		7.62			5.60			5.71		0
Republican voters		50.5			39.1			47.3		0
Urban population		89.3			88.2			74.7		0.22
Firearm ownership		28.4			18.1			31.3		0
Population over age 15 years		71.5			70.3			70.5		0
Black or African-American population		15.5			17.4			9.51		0
Violent crimes			770			521			363	0
Incarcerations			465			342			320	0
Homicides by year^f^										
1999			6.12			5.87			5.32	0.09
2000			5.87			5.65			5.02	0.02
2001			5.90			6.04			5.35	0.11
2002			6.04			5.80			5.42	0.25
2003			5.90			6.11			5.51	0
2004			5.93			5.50			5.00	0.08
2005			5.36			5.89			5.50	0.23

Abbreviations: V, variable importance weight.

^a^ Real: observed values from Florida. Synthetic: values from synthetic Florida (a weighted average of the 15 control states using the weights in Table 2, estimated using the synthetic control method).

^b^ Unweighted average of the 15 control states used in the synthetic control analysis.

^c^ Per 100,000 person-years.

^d^ The covariates reflect state-specific averages of yearly data from 1999 to 2004, except for unemployment, which was averaged using monthly observations from January 1999 to September 2005; urban population, which reflects a single measurement from 2000; and Republican voters, which is an average of the 2000 and 2004 US presidential elections. See Web Table 1 for data sources and details.

^e^ Gallons per capita, 21 years or older.

^f^ Monthly homicide rates per 100,000 population were averaged from January to December within each state and year (January to September in 2005). The rates are expressed per 100,000 person-years in the table.

Technically, the algorithm runs a nested optimization to also find the optimal variable importance weights that minimize the distance between the treated unit and synthetic control on a chosen set of pre-intervention outcomes (see Appendix to Abadie and Gardeazabal (11) for details). This set can be the entire pre-intervention time series (9) or a cross-validation subset of the pre-intervention period (12). Entering all pre-intervention outcomes into both the matching vector $\mathbf{X}$ and variable importance routine can lead to overfitting and will always result in variable importance weights that exclusively prioritize good fit on the pre-intervention outcomes over other covariates, and is hence not recommended (39). To avoid this issue, Abadie et al. (9) entered outcomes from 1975, 1980, and 1988 in their matching vector $\mathbf{X}$ and used yearly data from the entire “pre” period (1970–1988) to determine variable importance in a study of a tobacco tax reform in California on cigarette sales. Given that $\mathbf{X}$ contains outcome data from a subset of the time points used to determine variable importance, we refer to this strategy as a partial overlap approach. In a later paper examining the economic impacts of the reunification of Germany, they used data from 1971–1980 in the matching vector $\mathbf{X}$ (a training period) and used pre-intervention outcomes from 1981–1990 (validation period) to determine variable importance (i.e., a cross-validation approach) (12). This part of SCM is arguably the one where the analyst is given the greatest flexibility in terms of model specification. It is therefore important to present sensitivity analyses to demonstrate the robustness of the results to alternative specifications (see, e.g., Bonander (16) for examples) or to leave out a portion of the pre-intervention period for cross-validation to assess the quality of the model (21).

**Figure 2:**
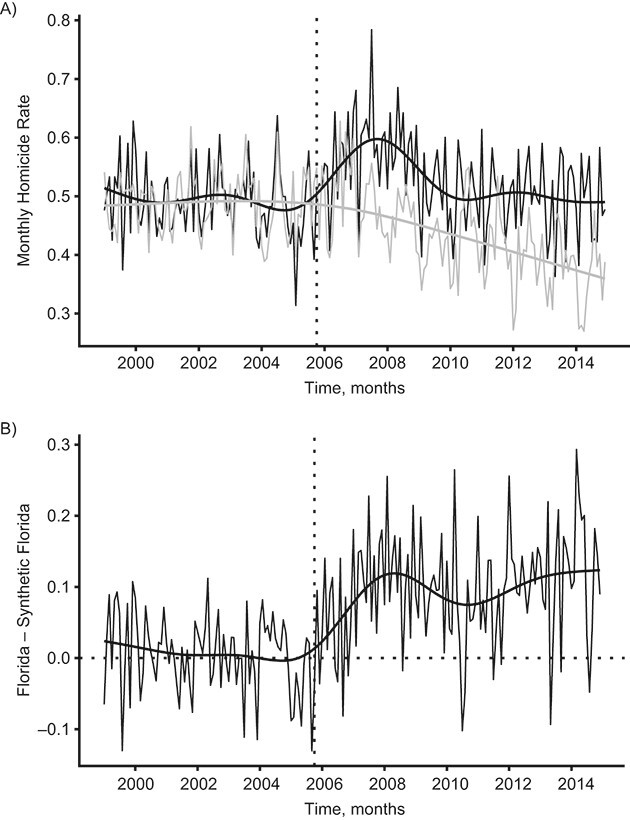
Outcomes and effect estimates from the synthetic control analysis. A) Homicide rates per 100,000 person-months in Florida (black lines) and synthetic Florida (gray lines) from January 1999 to December 2014. B) Pointwise difference in outcomes between Florida and synthetic Florida. The vertical line marks the implementation of Florida’s “stand your ground” law in October 2005. Predictions based on spline regression models were added to the plot after estimation to enhance visualization of the signal in the data and estimated effects.

In our example, we use a partial overlap approach and enter yearly averages of homicide rates from 1999–2005 into the matching vector $\mathbf{X}$ in addition to the covariates listed in Covariates, above, and use monthly homicide data from the entire pre-intervention period (January 1999 to September 2005) to determine variable importance. The resulting synthetic control for Florida consists primarily of New York, Maryland, New Jersey, and Massachusetts (their optimal unit weights, as determined by our SCM specification, are presented in Table 2).

## ASSESSING SYNTHETIC CONTROL FIT

The quality of the synthetic control can be assessed by examining the covariate balance between the treated unit and the synthetic control and how well the outcomes in the synthetic control unit fit those in the treated unit during the pre-intervention period.

### Balance check

Balance checks for synthetic controls are similar to those typically performed for covariate balancing methods (e.g., propensity score weighting) (10). We tabulate covariate values in Florida together with unweighted and weighted means from the donor pool in Table 3. We also include the variable importance weights produced by SCM for reference; greater imbalance is to be expected on covariates with low weights. In our case, SCM prioritizes balance on urbanicity in addition to the pre-intervention outcomes included in the matching vector.

### Pre-intervention fit

Pre-intervention fit can be assessed by plotting the time series of the observed outcomes in the treated unit versus the outcomes in the synthetic control (Figure 2A) or by plotting their difference (Figure 2B). In our example, the difference is concentrated around zero throughout the “pre” period, which implies a reasonably good fit. Systematic deviations from zero, such as diverging pre-intervention trends, would imply that there is cause for concern. Leaving out a portion of the pre-intervention period for cross-validation can also help assess the risk of bias. In our case, leaving out the last third of the period from the training period gives rise to similar results as the main analysis, and the synthetic control matches the treated unit closely in the left-out period (Web Figure 2).

## USING SYNTHETIC CONTROLS TO EVALUATE THE INTERVENTION

### Point estimation

Once a suitable specification for the synthetic control has been obtained, estimates can be calculated as any contrast between the postintervention outcomes in the treated unit and the synthetic control. Typical choices involve calculating time-specific differences and plotting the temporal evolution of the estimated effect (Figure 2B) or taking the difference or percentage change over the entire postintervention period. To obtain any of these, the first step is to calculate the estimated counterfactual for each postintervention time point *t*:

(2) \begin{equation*} \widehat{Y_{1t}(0)}=\sum \limits_{i=2}^N{Y}_{it}{w}_i^{\ast }, \end{equation*}

where ${Y}_{it}$ is the time-specific outcome in unit *i* (*i =* 1 is the treated unit, and the rest are controls), and ${w}_i^{\ast }$ is the unit weight assigned to each control unit by SCM. In our example, the average “post” period difference is 1.14 homicides per 100,000 person-years (equivalent to a 22% increase).

### Statistical inference

Obtaining valid inference statistics (i.e., *P* values and confidence intervals) can be challenging in panel data due to serial correlation (40), especially when there are few treated units (41). In large samples, one can typically use cluster-robust standard errors to handle this problem, but these methods tend to work poorly in small samples (42) (e.g., with fewer than 40 units, which is typical for SCM). Inference for SCM is further complicated by the constraints placed on the weights (43), which causes a type of bias that leads to nonnormal sampling distributions for effect estimates (44, 45) (regularization bias). As a consequence, it is difficult to derive general variance estimators (e.g., confidence intervals) for effect estimates based on SCM (43, 45). The typical alternative in such settings is to use the bootstrap, but this method tends to perform poorly with a single treated unit (22, 28). There are currently no best practice recommendations for how to perform statistical inference for SCM estimates, although the topic is an active area of research (43, 45–47).

**Figure 3:**
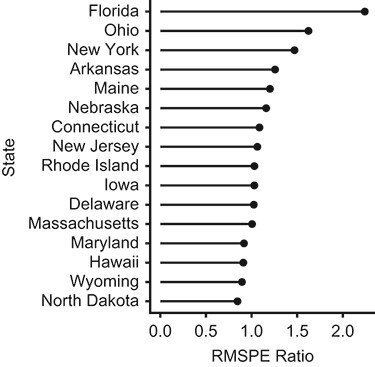
Results from the placebo analysis comparing estimated effects in Florida to placebo effects from other, untreated states. Size of the estimated (placebo) effects standardized by pre-intervention fit in all states in the data (ordered from largest to smallest). The data reflect the state-specific ratio between the postintervention and pre-intervention root mean squared prediction error (RMSPE). See text for further detail.

**Figure 4:**
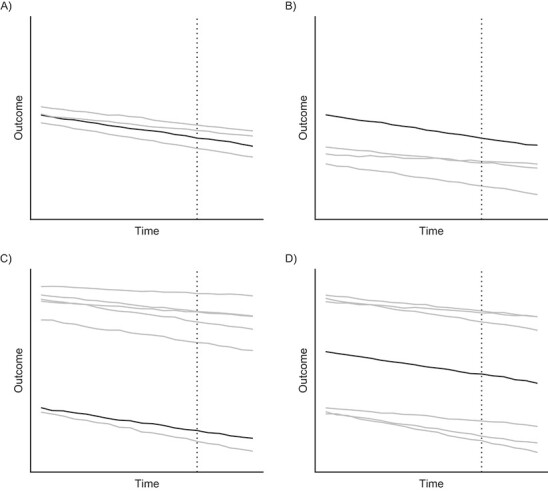
Toy data illustrating the potential appropriateness of the synthetic control method depending on the position of the treated unit compared with the units in the donor pool (“untreated”) with respect to the outcome. The vertical line marks the start of a hypothetical intervention. Each panel represents a different scenario: A) The treated unit and donor pool are similar (no reason to suspect interpolation bias with respect to the outcome dimension); B) the treated unit is outside the range of the pre-intervention outcomes in the donor pool (a synthetic control unit cannot be identified); C) the treated unit is within the range of the outcome data, but strong interpolation will be required from units with much higher outcomes (interpolation bias may occur); D) the treated unit is positioned at a void within the convex hull, and strong interpolation from units with both much lower and much higher pre-intervention outcomes will be required (interpolation bias may occur).

As an alternative, Abadie et al. (9) proposed assessing the significance of the estimated effects by estimating the effects of hypothetical interventions that “occur” at the same time as the intervention of interest in each control unit, and to compare these so-called “placebo effect” estimates with the actual effect estimate. This procedure is complicated by the fact that the quality of the pre-intervention fit may differ between each unit. However, the evidence for a causal effect is strengthened if the effect estimate is larger in the actual treated unit than in control units with placebo counterfactuals of comparable pre-intervention fit. To assess this, Abadie et al. (9) suggested quantifying a standardized and directionless effect measure that accounts for pre-intervention fit. The measure can be calculated as follows: For each control unit *i*, shift the treated unit into the control group and run an equivalent synthetic control analysis to the one in the treated unit and store the estimated counterfactuals $\widehat{Y_{it}(0)}$ (see equation 2). Then calculate the squared prediction error at each time point *t* by squaring the difference between the observed outcomes and the outcomes in the synthetic control in each unit (including the treated unit):

(3) \begin{equation*} {e}_{it}={\big({Y}_{it}-\widehat{Y_{it}(0)}\big)}^2 .\end{equation*}

Next, calculate the period-specific averages of ${e}_{it}$ for the pre-intervention and postintervention periods within each unit; let ${\overline{e}}_{i,{T}_0}$ and ${\overline{e}}_{i,{T}_1}$ denote these quantities, respectively. The standardized effect measure is then given by ${r}_i=\sqrt{{\overline{e}}_{i,{T}_1}}/\sqrt{{\overline{e}}_{i,{T}_0}}$ (the ratio between the postintervention and pre-intervention root mean squared prediction error), which gives a measure of the size of the effect relative to pre-intervention fit. The size of ${r}_i$ in the treated unit can then be compared with those based on the placebo studies among controls. After running this test on our data, we find that Florida has the largest ${r}_i$ of all states in the sample (Figure 3), which implies that it is unlikely to find an equally large effect estimate elsewhere in the data when the synthetic control analyses exhibit equivalent pre-intervention fit. This result can also be expressed as a permutation-based *P* value by dividing the rank of the treated unit (1 (the highest in Figure 3)) by the number of units in the data (16 in total): 1/16 = 0.0625 (see Abadie et al. (9) for a fuller explanation and Abadie (3) for additional discussion about the interpretation of these *P* values)).

We describe a recently proposed method for estimating confidence intervals for synthetic controls in Web Appendix 1 for readers who prefer conventional statistical inference (45). Our replication file contains R code to apply both approaches (20).

## OVERCOMING PRACTICAL AND METHODOLOGICAL ISSUES

This section describes common practical and methodological issues and offers recommendations to address them.

### The treated unit is outside the convex hull of the donor pool data

SCM only allows for interpolation within the empirical distribution of the control data. To construct a synthetic control that approximates the treated unit, the treated unit’s values on the variables included in the matching procedure must therefore fall within the range of the corresponding variables among the controls. Only allowing for interpolation prevents bias due to unrealistic estimates far from the empirical support of the data (48). In the SCM literature, this feature is typically referred to as the convex hull condition (3). While the condition extends to covariates, the biggest problems tend to arise if the treated unit has lower or higher values on the outcome variable than any of the available controls. The problem can be easily detected by inspecting the outcome data (Figure 4A; Figure 4B).

The convex hull condition can be relaxed by modifying the constraints on the unit weights (see, e.g., Bonander (17) and Doudchenko and Imbens (24)) or by subtracting the within-unit average of the pre-intervention outcomes from the time-specific outcomes in each unit (3). In the latter case, the algorithm will match on pre-intervention trends rather than trends and levels (similar to difference-in-differences estimation). It is then important to consider whether the outcome trend conveys the same information regardless of its level. See Abadie (3) and Hazlett and Xu (28) for further discussion about these modifications.

### Excessive interpolation

While SCM protects against extreme counterfactuals obtained via extrapolation, excessive interpolation may also be a cause for concern (9) (Figure 4C; Figure 4D). Again, it is advisable to inspect the raw data to analyze the degree of interpolation required to construct the synthetic control and—if deemed necessary—conduct sensitivity analyses to assess the robustness of the results to the exclusion of controls that differ greatly from the treated unit (12). Recent work by Abadie and L’Hour (30) provides another solution that involves penalizing dissimilar units in the optimization so that they are less likely to contribute to synthetic control.

### Noisy outcome data and overfitting

The original SCM algorithm was developed for time series that are measured with a minimal degree random fluctuations between time points (“noise”) (e.g., gross domestic product) (9). However, epidemiologic data may exhibit noise even in large populations (e.g., if the disease in question is rare). If SCM overfits to noise rather than the underlying trend, the results may be biased (49, 50). The data in our example may be characterized as noisy (Figure 2), and it is therefore important to assess whether our main results are sensitive to overfitting.

At least 2 strategies can be employed to handle noisy outcome data: filtering and de-biasing. Filtering involves trying to remove noise in the pre-intervention outcomes before SCM optimization (49, 51). In our case, the estimate changes from a 22% to 24% increase when we prefilter the pre-intervention outcomes using the method described by Fried (52). However, prefiltering the data adds another modeling step to the analysis that may increase the risk of misspecification bias. De-biasing instead involves subtracting an out-of-sample estimate of the bias from the effect estimate (44, 50). For details, see Web Appendix 1; the inference method described there uses de-biasing as a necessary step to construct valid confidence intervals.

### Additional robustness and falsification checks

Causal inference in SCM relies heavily on the absence of other confounding events in the postintervention period, including in the treated unit itself and in units that contribute to the synthetic control. It is therefore important to assess the risk of bias due to such events or other issues in the data. In Web Appendix 2, we discuss how placebo studies (12), manual restriction of the donor pool (15), and negative control outcomes (53) can be used to assess the robustness of the results in SCM studies.

## CONCLUDING REMARKS

We have presented a nontechnical tutorial to introduce SCM and discuss its main strengths and limitations. If used correctly, the method can provide valuable evidence about the effects of health interventions and policies (3, 6). We hope that this tutorial can raise awareness about SCM, including its limitations, and thereby enable more widespread and credible implementation of the method in epidemiologic research.

## Supplementary Material

Web_Material_kwab211
