# Supplementary material for: Synthetic Control Methods for the Evaluation of Single-Unit Interventions in Epidemiology: A Tutorial
Source: Am J Epidemiol. 2021 Aug 3;190(12):2700–11. doi: 10.1093/aje/kwab211 (PMC8634614; doi:10.1093/aje/kwab211)
Supplement: Web_Material_kwab211 [file web_material_kwab211.pdf]

WEB MATERIAL

Synthetic Control Methods for the Evaluation of Single-Unit Interventions in Epidemiology: A Tutorial

Carl Bonander, David Humphreys, Michelle Degli Esposti

Table of contents

Web Appendix 1: Confidence intervals for SCM.

Web Appendix 2: Additional robustness and falsification tests.

Web Figure 1. A simple logic model for ”stand your ground” (SYG) laws.

Web Figure 2. In-time placebo test leaving out the last third of the pre-intervention period.

Web Table 1. List of covariates used in the analysis.

Web Appendix 1: Confidence intervals for SCM

Deriving an analytical formula for the confidence intervals for synthetic controls has proven to be a difficult task, especially in settings when only a single unit is treated. The topic is an active area of research. We here present one alternative that was recently proposed by Chernozhukov *et al*[1]. The method is fairly easy to implement and appears to have desirable finite sample properties according to extensive simulations[1,2]. Our exposition below follows the descriptions in Chernozhukov *et al* closely, which focuses on estimating confidence intervals for absolute effects. Bonander[2] describes and demonstrates how the approach can be applied to estimate confidence intervals for rate ratios. We refer to the original publication for additional details and proofs[1]. An example using R is also available in the replication code for the paper (https://osf.io/6udsq/).

Non-technical summary

The approach builds on the recent literature on statistical inference with machine learning estimators, which rely on sample splitting and cross-fitting to adjust for regularization bias[3] (effectively recovering a normal sampling distribution for data-driven effect estimators). It also has desirable properties such as robustness to autocorrelation and reasonable confidence interval coverage rates in small samples according to simulations[1,2]. The method relies on the cross-fitting of two or more synthetic controls estimated while holding out different portions of the pre-intervention data for cross-validation. The mean difference between the outcomes in the treated unit and the synthetic control in the holdout periods is then used as an estimate of the bias of the effect estimates. The estimated bias is then subtracted from the actual effect estimates in the post-intervention period, which allows for the construction of CIs using a *t*-distribution. Applying this method to the “stand your ground” law example from the main body of the paper using three holdout samples, we obtain a bias-corrected effect estimate of 1.01 (95% CI: 0.14, 1.88) homicides per 100.000 person-years (equivalent to a 19% increase).

Technical details

First, we define the estimator for the causal effect on the full post-treatment period as:

$$\hat{\tau} = \left(\frac{1}{T_1}\sum_{t=T_0+1}^{T} Y_{1t}\right) - \left(\frac{1}{T_1}\sum_{t=T_0+1}^{T}\sum_{i=2}^{N} Y_{it} w_i^*\right) \tag{A.1}$$

where w_i^* are the synthetic control weights; T is the number of time points in the data; T_1 is the number of time points in the post-intervention period; T_0 reflects the number of time points in the pre-intervention period, and N is the number of units in the data (where i = 1 is the treated unit and the rest are controls). We should generally expect this estimator to have an asymptotically non-normal distribution due to the regularization required for estimation of the high-dimensional weight parameter[3]. Chernozhukov *et al* propose a *K*-fold cross-fitting procedure to control to achieve asymptotic normality[1]. The de-biasing step may also help control for bias due to overfitting[3]. The procedure can be summarized as follows. First, choose *K* consecutive blocks from the pre-treatment period: $H_1 \cup H_2 \cup \ldots \cup H_k \subseteq \{1, \ldots, T_0\}$. Next, define $r = \min\{[T_0/K], T_1\}$, and let $H_k = \{(k-1)r + 1, \ldots, kr\}$ for $1 \leq k \leq K$. If T_0/K is not integer-valued, we round it down to the nearest integer.

Now, let $w_{i(k)}^*$ denote the synthetic control weight for unit *i* obtained when the period H_k is left out of the estimation procedure. Further, let $|H_k|$ denote the length of the *k*th holdout sample. The debiased estimate for the *k*th holdout sample is then given by

$$\hat{\tau}_k = \left[\left(\frac{1}{T_1}\sum_{t=T_0+1}^{T} Y_{1t}\right) - \left(\frac{1}{T_1}\sum_{t=T_0+1}^{T}\sum_{i=2}^{N} Y_{it} w_{i(k)}^*\right)\right] - \left[\left(\frac{1}{|H_k|}\sum_{t\in H_k} Y_{1t}\right) - \left(\frac{1}{|H_k|}\sum_{t\in H_k}\sum_{i=2}^{N} Y_{it} w_{i(k)}^*\right)\right] \tag{A.2}$$

The final cross-fitted estimator of the average post-intervention effect is given by

$$\hat{\tau}^{CF} = \frac{1}{K}\sum_{k=1}^{K} \hat{\tau}_k \tag{A.3}$$

Finally, Chernozhukov *et al* derive a test statistic to produce confidence intervals for the effect estimate using a t-distribution[1]:

$$CI_K(1-\alpha) = \left[\hat{\tau}^{CF} - t_{K-1}\left(1-\frac{\alpha}{2}\right)\frac{\hat{\sigma}_k}{\sqrt{K}}, \hat{\tau}^{CF} + t_{K-1}\left(1-\frac{\alpha}{2}\right)\frac{\hat{\sigma}_k}{\sqrt{K}}\right] \tag{A.4}$$

where

$$\hat{\sigma}_k = \sqrt{1+\frac{Kr}{T_1}}\sqrt{\frac{1}{K-1}\sum_{k=1}^{K}(\hat{\tau}_k - \hat{\tau}^{CF})^2} \tag{A.5}$$

and $t_{K-1}(1-\alpha/2)$ is the $(1-\alpha/2)$-quantile of a student *t*-distribution with $K-1$ degrees of freedom.

We note that the choice of K will affect the width of the confidence interval, which is a clear drawback to the test. Chernozhukov *et al* investigate $K=2$ and $K=3$ using extensive simulations, finding that $K=2$ has better coverage rates when the pre-intervention period is short (15 time points in their case)[1]. When the period is long (say, 300 time points as in their second simulation), both choices appear to provide reasonable coverage. They conclude that $K=3$ seems to be a reasonable trade-off between average confidence interval width (i.e., efficiency) and coverage accuracy based on extensive simulations.

Web Appendix 2: Additional robustness and falsification tests

This in Appendix, we detail a few strategies that can be employed to assess the risk of bias in SCM studies.

Placebo studies

One strategy for probing the risk of bias is to conduct placebo studies as discussed in the main text (section on *Statistical Inference*); artificially re-assigning the intervention to untreated units in the data can help identify issues in the data or estimation procedure. If the pre-intervention period sufficiently long, Abadie *et al* also suggest conducting in-time placebo studies[4] (e.g., shifting the intervention date to 10 years earlier to assess if SCM identifies an effect during a period when there should be none).

Restricting the donor pool

Manually restricting the donor pool may sometimes help reduce the risk of bias due to known events that are difficult for the SCM algorithm to predict using pre-intervention data alone. For example, if the treated unit experiences other unexpected events that affect the outcome in the post-intervention period (say, the opioid epidemic), it may be appropriate to restrict the donor pool to units that would have likely been affected similarly by these events in absence of the intervention[5]. Removing units that contribute strongly to the synthetic control (e.g., New York in our empirical example) may also serve as a robustness check to investigate whether the estimates are driven entirely by the inclusion of specific controls[6].

Negative controls

The use of negative control outcomes can also help assess the sensitivity of the results to confounding events in the post-intervention period, provided that they are affected by these events in the same manner as the actual outcome[7]. For instance, re-running the analyses in our example with suicides by firearm as a negative control outcome yields an effect estimate close to zero (0.15 [95% CI: -0.89, 1.20] per 100.000 person-years; 2% increase). This indicates that the impact is isolated to homicides and cannot be explained by other changes that affect firearm mortality in general. However, it does not rule out the possibility for biases that are isolated to homicide rates.

Web Figures

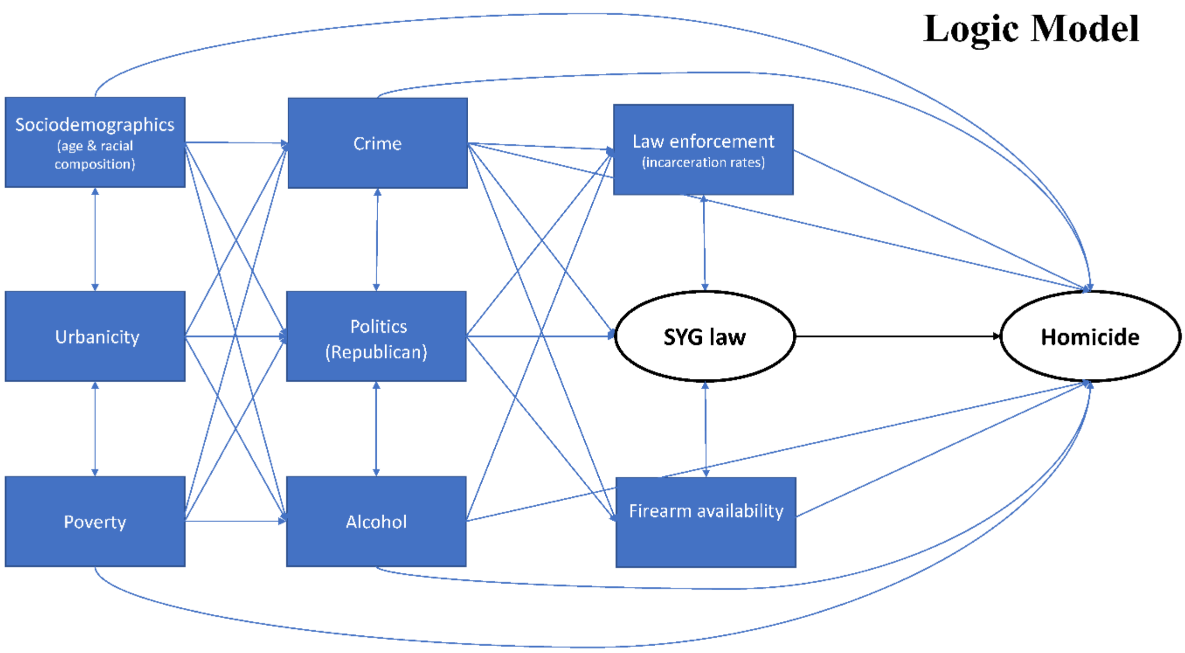

Web Figure 1. A simple logic model for ”stand your ground” (SYG) laws, with hypothesized confounding factors (blue).

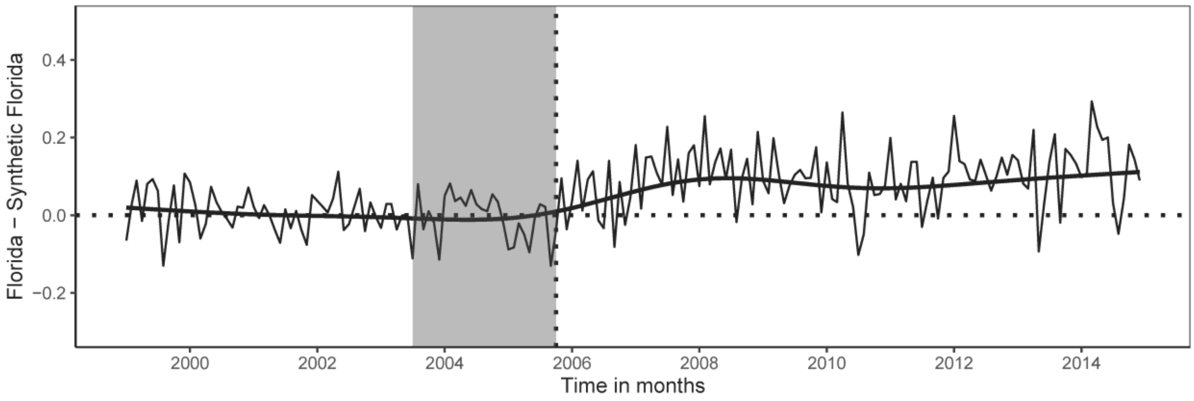

Web Figure 2. Difference in homicide rates per 100.000 person-months between Florida and Synthetic Florida when leaving out the final third of the pre-intervention period to serve as an in-time placebo period (average placebo effect estimate [shaded area]: -0.19 per 100.000 person-years, average post-intervention effect estimate [after dashed vertical line]: 0.97 per 100.000 person-years). Estimates from a spline regression model have been added to the plot after estimation to enhance the signal in the estimated effects.

Web Table

Web Table 1. List of covariates (n=9) for the synthetic control analysis based on the logic model for “stand your ground” laws.

Theoretical construct[a]	Measure	Data collection intervals	Source
**Socio-demographics**	- Age composition (proportion over 15y)<br>- Racial composition (proportion Black or African American)	Year	U.S. Census Bureau
**Urbanicity**	Percentage of the population living in urban areas	Every 10 years (from 2000)	U.S. Census Bureau
**Poverty**	Unemployment rates (adjusted)	Month	U.S. Bureau of Labor Statistics
**Alcohol**	Gallons of ethanol consumption per capita (21 years and older)[b]	Year	National Institute on Alcohol Abuse and Alcoholism
**Crime**	Violent crime rates (per 100,000 population)[b]	Year	FBI Uniform Crime Reports
**Law enforcement**	Incarceration rates (per 100,000 population)	Year	U.S. Department of Justice
**Firearm availability**	Estimated firearm ownership rate (%)[b]	Year	Schell et al: https://doi.org/10.7249/TL354
**Politics**	Proportion of Republican votes out of total voters	Every 4 years (from 2000)	U.S. Federal Election Commission

a Cross-reference with Logic Model (Web Figure 1).

b Using updated data not in original publications.

References

1. Chernozhukov V, Wuthrich K, Zhu Y. Practical and robust t-test based inference for synthetic control and related methods. *arXiv:181210820 [econ]*, http://arxiv.org/abs/1812.10820 (2019, accessed 16 November 2019).

2. Bonander C. A (Flexible) Synthetic Control Method for Count Data and Other Non-Negative Outcomes. *Epidemiology*; [available online ahead of print Jun 25, 2021]. Epub ahead of print 2021. DOI: 10.1097/ede.0000000000001388.

3. Chernozhukov V, Chetverikov D, Demirer M, et al. Double/debiased machine learning for treatment and structural parameters. *The Econometrics Journal* 2018; 21: C1–C68.

4. Abadie A, Diamond A, Hainmueller J. Comparative Politics and the Synthetic Control Method. *American Journal of Political Science* 2015; 59: 495–510.

5. Degli Esposti M, Spreckelsen T, Gasparrini A, et al. Can synthetic controls improve causal inference in interrupted time series evaluations of public health interventions? *International Journal of Epidemiology* 2021; 49: 2010–2020.

6. Bonander C. Compared with what? Estimating the effects of injury prevention policies using the synthetic control method. *Inj Prev* 2018; 24: i60–i66.

7. Lipsitch M, Tchetgen ET, Cohen T. Negative Controls: A Tool for Detecting Confounding and Bias in Observational Studies. *Epidemiology* 2010; 21: 383–388.
